# *cis*-Dioxido[*N*′-(2-oxidobenzyl­idene)pyridinium-4-carbohydrazidato-κ^3^ *O*,*N*′,*O*′]vanadium(V)

**DOI:** 10.1107/S1600536812003637

**Published:** 2012-02-04

**Authors:** Gholam Hossein Shahverdizadeh, Seik Weng Ng, Edward R. T. Tiekink, Babak Mirtamizdoust

**Affiliations:** a Department of Chemistry, Faculty of Science, Tabriz Branch, Islamic Azad University, PO Box 1655, Tabriz, Iran; b Department of Chemistry, University of Malaya, 50603 Kuala Lumpur, Malaysia; c Chemistry Department, Faculty of Science, King Abdulaziz University, PO Box 80203, Jeddah, Saudi Arabia; d Department of Inorganic Chemistry, Faculty of Chemistry, University of Tabriz, PO Box 5166616471, Tabriz, Iran

## Abstract

The title Schiff base complex, [V(C_13_H_10_N_3_O_2_)O_2_], features a square-pyramidal coordination geometry defined by the *O*,*N*′,*O*′-donors of the tridentate Schiff base ligand and two oxide O atoms; one oxide O atom occupies the apical position. In the crystal, pyridinium–oxide N—H⋯O hydrogen bonds lead to zigzag supra­molecular chains with a flattened topology along [101]. The investigated crystal was twinned by nonmerohedry; the minor component refined to 18.5 (5)%.

## Related literature

For a related Schiff base vanadyl complex containing a protonated pyridyl residue, see: Yu *et al.* (2007 ▶). For the crystallization procedure, see: Harrowfield *et al.* (1996 ▶). For a related structure, see: Shahverdizadeh *et al.* (2012 ▶). For additional structural analysis, see: Spek (2009 ▶); Addison *et al.* (1984 ▶).
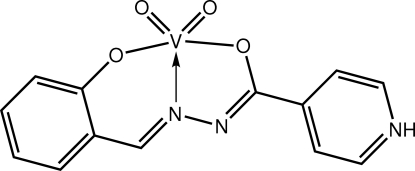


## Experimental

### 

#### Crystal data

[V(C_13_H_10_N_3_O_2_)O_2_]
*M* *_r_* = 323.18
Monoclinic, 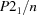

*a* = 7.1215 (3) Å
*b* = 14.5243 (6) Å
*c* = 11.9233 (5) Å
β = 94.081 (3)°
*V* = 1230.16 (9) Å^3^
*Z* = 4
Cu *K*α radiation
μ = 6.96 mm^−1^
*T* = 100 K
0.25 × 0.25 × 0.25 mm

#### Data collection

Agilent SuperNova Dual diffractometer with Atlas detector
Absorption correction: multi-scan (*CrysAlis PRO*; Agilent, 2010 ▶) *T*_min_ = 0.275, *T*_max_ = 0.275
2559 measured reflections
2559 independent reflections
2467 reflections with *I* > 2σ(*I*)

#### Refinement

*R*[*F* ^2^ > 2σ(*F* ^2^)] = 0.064
*wR*(*F* ^2^) = 0.171
*S* = 1.26
2559 reflections
191 parameters
H-atom parameters constrained
Δρ_max_ = 1.36 e Å^−3^
Δρ_min_ = −0.84 e Å^−3^

### 

Data collection: *CrysAlis PRO* (Agilent, 2010 ▶); cell refinement: *CrysAlis PRO*; data reduction: *CrysAlis PRO*; program(s) used to solve structure: *SHELXS97* (Sheldrick, 2008 ▶); program(s) used to refine structure: *SHELXL97* (Sheldrick, 2008 ▶); molecular graphics: *ORTEP-3* (Farrugia, 1997 ▶) and *DIAMOND* (Brandenburg, 2006 ▶); software used to prepare material for publication: *publCIF* (Westrip, 2010 ▶).

## Supplementary Material

Crystal structure: contains datablock(s) global, I. DOI: 10.1107/S1600536812003637/bt5805sup1.cif

Structure factors: contains datablock(s) I. DOI: 10.1107/S1600536812003637/bt5805Isup2.hkl

Additional supplementary materials:  crystallographic information; 3D view; checkCIF report

## Figures and Tables

**Table 1:** Selected bond lengths (Å)

V—O1	1.904 (3)
V—O2	2.006 (3)
V—O3	1.610 (4)
V—O4	1.654 (3)
V—N1	2.140 (4)

**Table 2:** Hydrogen-bond geometry (Å, °)

*D*—H⋯*A*	*D*—H	H⋯*A*	*D*⋯*A*	*D*—H⋯*A*
N3—H1⋯O4^i^	0.88	1.75	2.610 (5)	164

Symmetry code: (i) 
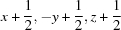
.

**Fig. 1.**
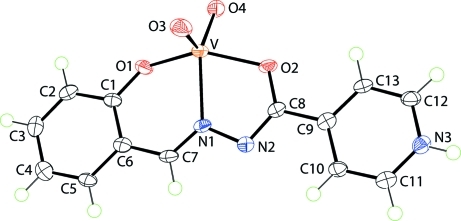
The molecular structure of (I) showing the atom-labelling scheme and displacement ellipsoids at the 70% probability level.

**Fig. 2.**
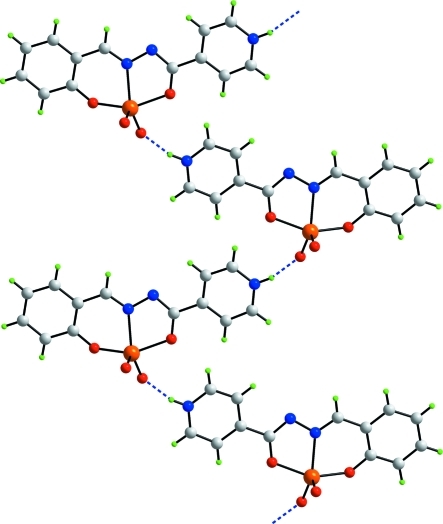
A view of the zigzag supramolecular chain along [101] in (I). The N—H···O hydrogen bonds are shown as blue dashed lines.

## supplementary crystallographic information

### Comment

In continuation of structural studies of vanadyl Schiff base complexes (Shahverdizadeh *et al.*, 2012), the title complex, (I), was characterized.

The V atom in (I) is coordinated by the *O*,*N*,*O*-tridentate Schiff base ligand and two oxido-O atoms. The resulting NO_4_ donor set is based on a square pyramid with oxido-O3 atom in the axial position. The coordination geometry is quantified by the calculation of τ = 0.05 which compares with τ = 0.0 for an ideal square pyramidal geometry and τ = 1.0 for an ideal trigonal bipyramid (Addison *et al.*, 1984). The V═O3 bond length is significantly shorter than the V═O4 bond length, Table 1, an observation ascribed to the influence exerted by the *trans*-N1 atom and the participation of the oxido O4 atom in hydrogen bonding, Table 2. The pyridinium-NH···O(oxido) hydrogen bonding leads to a zigzag chain along [101] with a flattened topology.

The Schiff base ligand in (I) is present as a pyridinium cation. A precedent exists in the literature in a closely related V complex (Yu *et al.*, 2007).

### Experimental

A solution of salicylaldehyde (10 mmol) in EtOH (25 ml) was added drop-wise to the solution of 4-pyridinecarboxylic acid hydrazide (10 mmol) in EtOH (15 ml). The mixture was refluxed for 8 h. The yellow precipitate was removed by filtration and recrystallized from MeOH solution. The product (0.5 mmol) was placed in one arm of a branched tube (Harrowfield *et al.*, 1996) and vanadium(IV) oxide acetylacetonate (0.5 mmol) in the other. Methanol was then added to fill both arms, the tube sealed and the ligand-containing arm immersed in a bath at 333 K, while the other was left at ambient temperature. After 8 d, crystals had deposited in the arm held at ambient temperature. These filtered off, washed with acetone and ether, and air-dried. Yield: 72%. M.pt. 566 K.

### Refinement

The crystal was a non-merohedral twin. The twin components were separated by the *TwinRotMat* routine in *PLATON* (Spek, 2009); the minor component refined to 18.5 (5)%. Carbon-bound H-atoms were placed in calculated positions [C—H 0.95 Å, *U*_iso_(H) 1.2*U*_eq_(C)] and were included in the refinement in the riding model approximation. The pyridinium H-atom was located in a difference Fourier map and was refined with a distance restraint of N—H 0.88±0.01 Å with *U*_iso_(H) 1.2*U*_eq_(N). The maximum and minimum residual electron density peaks of 1.36 and 0.84 e Å^-3^, respectively, were located 0.91 Å and 0.65 Å from the V atom, respectively. Owing to poor agreement, the reflection (4 8 11) was omitted from the final refinement.

### Crystal data

**Table:**

[V(C_13_H_10_N_3_O_2_)O_2_]	*F*(000) = 656
*M**_r_* = 323.18	*D*_x_ = 1.745 Mg m^−^^3^
Monoclinic, *P*2_1_/*n*	Cu *K*α radiation, λ = 1.54184 Å
Hall symbol: -P 2yn	Cell parameters from 5747 reflections
*a* = 7.1215 (3) Å	θ = 3.0–76.6°
*b* = 14.5243 (6) Å	µ = 6.96 mm^−^^1^
*c* = 11.9233 (5) Å	*T* = 100 K
β = 94.081 (3)°	Polyhedron, brown
*V* = 1230.16 (9) Å^3^	0.25 × 0.25 × 0.25 mm
*Z* = 4	

### Data collection

**Table:**

Agilent SuperNova Dual diffractometer with Atlas detector	2559 independent reflections
Radiation source: SuperNova (Cu) X-ray Source	2467 reflections with *I* > 2σ(*I*)
Mirror monochromator	*R*_int_ = 0.000
Detector resolution: 10.4041 pixels mm^-1^	θ_max_ = 76.8°, θ_min_ = 4.8°
ω scans	*h* = −8→8
Absorption correction: multi-scan (*CrysAlis PRO*; Agilent, 2010)	*k* = −18→18
*T*_min_ = 0.275, *T*_max_ = 0.275	*l* = −1→14
2559 measured reflections	

### Refinement

**Table:**

Refinement on *F*^2^	Primary atom site location: structure-invariant direct methods
Least-squares matrix: full	Secondary atom site location: difference Fourier map
*R*[*F*^2^ > 2σ(*F*^2^)] = 0.064	Hydrogen site location: inferred from neighbouring sites
*wR*(*F*^2^) = 0.171	H-atom parameters constrained
*S* = 1.26	*w* = 1/[σ^2^(*F*_o_^2^) + (0.0278*P*)^2^ + 7.6276*P*] where *P* = (*F*_o_^2^ + 2*F*_c_^2^)/3
2559 reflections	(Δ/σ)_max_ < 0.001
191 parameters	Δρ_max_ = 1.36 e Å^−^^3^
0 restraints	Δρ_min_ = −0.84 e Å^−^^3^

### Special details

**Table:**

Geometry. All s.u.'s (except the s.u. in the dihedral angle between two l.s. planes) are estimated using the full covariance matrix. The cell s.u.'s are taken into account individually in the estimation of s.u.'s in distances, angles and torsion angles; correlations between s.u.'s in cell parameters are only used when they are defined by crystal symmetry. An approximate (isotropic) treatment of cell s.u.'s is used for estimating s.u.'s involving l.s. planes.
Refinement. Refinement of *F*^2^ against ALL reflections. The weighted *R*-factor *wR* and goodness of fit *S* are based on *F*^2^, conventional *R*-factors *R* are based on *F*, with *F* set to zero for negative *F*^2^. The threshold expression of *F*^2^ > 2σ(*F*^2^) is used only for calculating *R*-factors(gt) *etc*. and is not relevant to the choice of reflections for refinement. *R*-factors based on *F*^2^ are statistically about twice as large as those based on *F*, and *R*-factors based on ALL data will be even larger.

### Fractional atomic coordinates and isotropic or equivalent isotropic displacement parameters (Å^2^)

**Table:**

	*x*	*y*	*z*	*U*_iso_*/*U*_eq_	
V	0.65274 (12)	0.54174 (6)	0.30415 (6)	0.0148 (2)	
O1	0.5622 (5)	0.6639 (2)	0.3213 (3)	0.0182 (7)	
O2	0.6790 (5)	0.4125 (2)	0.3624 (3)	0.0153 (7)	
O3	0.8525 (5)	0.5579 (3)	0.2519 (3)	0.0236 (8)	
O4	0.4995 (5)	0.5080 (2)	0.2016 (3)	0.0203 (8)	
N2	0.7647 (6)	0.4717 (3)	0.5387 (3)	0.0153 (8)	
N1	0.7142 (6)	0.5531 (3)	0.4819 (3)	0.0131 (8)	
N3	0.9037 (6)	0.1409 (3)	0.5891 (3)	0.0160 (8)	
H1	0.9366	0.0860	0.6149	0.019*	
C1	0.6069 (7)	0.7323 (3)	0.3920 (4)	0.0135 (9)	
C2	0.5699 (7)	0.8232 (3)	0.3575 (4)	0.0162 (9)	
H2	0.5206	0.8347	0.2827	0.019*	
C3	0.6035 (7)	0.8964 (3)	0.4303 (4)	0.0186 (10)	
H3	0.5744	0.9573	0.4055	0.022*	
C4	0.6804 (7)	0.8817 (3)	0.5407 (4)	0.0180 (10)	
H4	0.7032	0.9319	0.5907	0.022*	
C5	0.7221 (7)	0.7929 (3)	0.5749 (4)	0.0146 (9)	
H5	0.7756	0.7825	0.6491	0.018*	
C6	0.6874 (6)	0.7174 (3)	0.5027 (4)	0.0135 (9)	
C7	0.7303 (7)	0.6258 (3)	0.5432 (4)	0.0146 (9)	
H7	0.7731	0.6187	0.6201	0.018*	
C8	0.7437 (7)	0.4041 (3)	0.4673 (4)	0.0140 (9)	
C9	0.7986 (6)	0.3110 (3)	0.5094 (4)	0.0135 (9)	
C10	0.8734 (6)	0.2998 (3)	0.6209 (4)	0.0144 (9)	
H10	0.8878	0.3512	0.6700	0.017*	
C11	0.9251 (7)	0.2134 (3)	0.6575 (4)	0.0158 (9)	
H11	0.9768	0.2051	0.7325	0.019*	
C12	0.8337 (7)	0.1491 (3)	0.4826 (4)	0.0165 (10)	
H12	0.8204	0.0959	0.4363	0.020*	
C13	0.7805 (7)	0.2341 (3)	0.4394 (4)	0.0147 (9)	
H13	0.7326	0.2402	0.3633	0.018*	

### Atomic displacement parameters (Å^2^)

**Table:**

	*U*^11^	*U*^22^	*U*^33^	*U*^12^	*U*^13^	*U*^23^
V	0.0189 (4)	0.0164 (4)	0.0089 (4)	−0.0021 (3)	0.0006 (3)	−0.0011 (3)
O1	0.0264 (19)	0.0157 (16)	0.0122 (16)	0.0027 (14)	−0.0009 (14)	−0.0033 (13)
O2	0.0190 (17)	0.0179 (16)	0.0084 (14)	0.0027 (14)	−0.0028 (12)	0.0011 (12)
O3	0.028 (2)	0.0260 (19)	0.0183 (17)	−0.0065 (16)	0.0091 (15)	−0.0051 (14)
O4	0.028 (2)	0.0172 (16)	0.0151 (17)	−0.0034 (15)	−0.0057 (14)	0.0013 (13)
N2	0.018 (2)	0.0150 (19)	0.0125 (18)	−0.0010 (16)	−0.0001 (15)	0.0001 (15)
N1	0.0134 (19)	0.0172 (19)	0.0090 (17)	−0.0012 (15)	0.0024 (14)	0.0010 (14)
N3	0.016 (2)	0.0169 (19)	0.0153 (19)	0.0043 (16)	0.0030 (15)	0.0024 (15)
C1	0.011 (2)	0.018 (2)	0.012 (2)	0.0004 (17)	0.0019 (17)	−0.0018 (17)
C2	0.015 (2)	0.022 (2)	0.012 (2)	0.0002 (19)	0.0031 (17)	0.0031 (18)
C3	0.018 (2)	0.014 (2)	0.025 (3)	0.0009 (19)	0.007 (2)	0.0022 (19)
C4	0.014 (2)	0.018 (2)	0.022 (2)	−0.0007 (19)	0.0019 (19)	−0.0026 (19)
C5	0.015 (2)	0.018 (2)	0.011 (2)	0.0007 (18)	0.0040 (17)	−0.0035 (17)
C6	0.009 (2)	0.019 (2)	0.013 (2)	−0.0009 (17)	0.0049 (17)	−0.0017 (17)
C7	0.015 (2)	0.021 (2)	0.008 (2)	−0.0014 (19)	0.0021 (17)	−0.0007 (17)
C8	0.013 (2)	0.018 (2)	0.012 (2)	−0.0003 (18)	0.0014 (17)	0.0021 (17)
C9	0.007 (2)	0.019 (2)	0.015 (2)	0.0000 (17)	0.0023 (16)	0.0018 (18)
C10	0.012 (2)	0.018 (2)	0.012 (2)	−0.0026 (18)	−0.0020 (17)	0.0000 (17)
C11	0.013 (2)	0.021 (2)	0.013 (2)	0.0009 (18)	0.0011 (17)	0.0012 (18)
C12	0.019 (2)	0.020 (2)	0.011 (2)	0.0035 (19)	0.0031 (18)	−0.0005 (18)
C13	0.016 (2)	0.019 (2)	0.009 (2)	−0.0001 (18)	0.0025 (17)	0.0017 (17)

### Geometric parameters (Å, º)

**Table:**

V—O1	1.904 (3)	C3—C4	1.405 (7)
V—O2	2.006 (3)	C3—H3	0.9500
V—O3	1.610 (4)	C4—C5	1.379 (7)
V—O4	1.654 (3)	C4—H4	0.9500
V—N1	2.140 (4)	C5—C6	1.405 (6)
O1—C1	1.327 (6)	C5—H5	0.9500
O2—C8	1.307 (5)	C6—C7	1.441 (7)
N2—C8	1.301 (6)	C7—H7	0.9500
N2—N1	1.396 (5)	C8—C9	1.485 (6)
N1—C7	1.285 (6)	C9—C13	1.394 (7)
N3—C11	1.335 (6)	C9—C10	1.406 (6)
N3—C12	1.336 (6)	C10—C11	1.370 (7)
N3—H1	0.8800	C10—H10	0.9500
C1—C2	1.402 (7)	C11—H11	0.9500
C1—C6	1.418 (6)	C12—C13	1.382 (7)
C2—C3	1.383 (7)	C12—H12	0.9500
C2—H2	0.9500	C13—H13	0.9500
			
O3—V—O4	108.16 (19)	C5—C4—H4	120.6
O3—V—O1	102.77 (18)	C3—C4—H4	120.6
O4—V—O1	98.34 (16)	C4—C5—C6	121.5 (4)
O3—V—O2	101.95 (17)	C4—C5—H5	119.2
O4—V—O2	91.08 (15)	C6—C5—H5	119.2
O1—V—O2	149.21 (14)	C5—C6—C1	119.6 (4)
O3—V—N1	104.35 (17)	C5—C6—C7	119.4 (4)
O4—V—N1	146.37 (17)	C1—C6—C7	121.0 (4)
O1—V—N1	82.48 (15)	N1—C7—C6	124.0 (4)
O2—V—N1	73.82 (14)	N1—C7—H7	118.0
C1—O1—V	134.3 (3)	C6—C7—H7	118.0
C8—O2—V	115.9 (3)	N2—C8—O2	124.9 (4)
C8—N2—N1	108.0 (4)	N2—C8—C9	116.8 (4)
C7—N1—N2	114.2 (4)	O2—C8—C9	118.3 (4)
C7—N1—V	129.1 (3)	C13—C9—C10	119.1 (4)
N2—N1—V	116.3 (3)	C13—C9—C8	121.0 (4)
C11—N3—C12	122.0 (4)	C10—C9—C8	119.9 (4)
C11—N3—H1	119.0	C11—C10—C9	118.8 (4)
C12—N3—H1	119.0	C11—C10—H10	120.6
O1—C1—C2	119.1 (4)	C9—C10—H10	120.6
O1—C1—C6	122.7 (4)	N3—C11—C10	120.8 (4)
C2—C1—C6	118.2 (4)	N3—C11—H11	119.6
C3—C2—C1	121.3 (4)	C10—C11—H11	119.6
C3—C2—H2	119.3	N3—C12—C13	120.5 (4)
C1—C2—H2	119.3	N3—C12—H12	119.7
C2—C3—C4	120.6 (5)	C13—C12—H12	119.7
C2—C3—H3	119.7	C12—C13—C9	118.8 (4)
C4—C3—H3	119.7	C12—C13—H13	120.6
C5—C4—C3	118.8 (4)	C9—C13—H13	120.6
			
O3—V—O1—C1	−73.7 (4)	C4—C5—C6—C7	−178.8 (5)
O4—V—O1—C1	175.5 (4)	O1—C1—C6—C5	−176.7 (4)
O2—V—O1—C1	69.0 (6)	C2—C1—C6—C5	1.9 (7)
N1—V—O1—C1	29.4 (4)	O1—C1—C6—C7	1.9 (7)
O3—V—O2—C8	92.1 (3)	C2—C1—C6—C7	−179.6 (4)
O4—V—O2—C8	−159.1 (3)	N2—N1—C7—C6	179.6 (4)
O1—V—O2—C8	−50.7 (5)	V—N1—C7—C6	7.4 (7)
N1—V—O2—C8	−9.6 (3)	C5—C6—C7—N1	−175.7 (5)
C8—N2—N1—C7	−179.4 (4)	C1—C6—C7—N1	5.7 (7)
C8—N2—N1—V	−6.2 (5)	N1—N2—C8—O2	−2.6 (6)
O3—V—N1—C7	82.3 (4)	N1—N2—C8—C9	177.3 (4)
O4—V—N1—C7	−112.8 (5)	V—O2—C8—N2	10.6 (6)
O1—V—N1—C7	−19.1 (4)	V—O2—C8—C9	−169.3 (3)
O2—V—N1—C7	−179.2 (4)	N2—C8—C9—C13	179.7 (4)
O3—V—N1—N2	−89.8 (3)	O2—C8—C9—C13	−0.4 (7)
O4—V—N1—N2	75.1 (4)	N2—C8—C9—C10	−1.5 (7)
O1—V—N1—N2	168.9 (3)	O2—C8—C9—C10	178.3 (4)
O2—V—N1—N2	8.7 (3)	C13—C9—C10—C11	−0.5 (7)
V—O1—C1—C2	154.5 (4)	C8—C9—C10—C11	−179.2 (4)
V—O1—C1—C6	−27.0 (7)	C12—N3—C11—C10	0.8 (7)
O1—C1—C2—C3	176.1 (4)	C9—C10—C11—N3	−0.5 (7)
C6—C1—C2—C3	−2.5 (7)	C11—N3—C12—C13	0.0 (7)
C1—C2—C3—C4	1.5 (7)	N3—C12—C13—C9	−1.0 (7)
C2—C3—C4—C5	0.2 (7)	C10—C9—C13—C12	1.2 (7)
C3—C4—C5—C6	−0.8 (7)	C8—C9—C13—C12	179.9 (4)
C4—C5—C6—C1	−0.3 (7)		

### Hydrogen-bond geometry (Å, º)

**Table:**

*D*—H···*A*	*D*—H	H···*A*	*D*···*A*	*D*—H···*A*
N3—H1···O4^i^	0.88	1.75	2.610 (5)	164

Symmetry code: (i) *x*+1/2, −*y*+1/2, *z*+1/2.
